# Survival of patients with cancers of the female genital organs in Poland, 2000–2019

**DOI:** 10.1038/s41598-023-35749-6

**Published:** 2023-05-25

**Authors:** Florentino Luciano Caetano dos Santos, Urszula Wojciechowska, Irmina Maria Michalek, Joanna Didkowska

**Affiliations:** Polish National Cancer Registry, Maria Sklodowska-Curie National Research Institute of Oncology, Ul. Wawelska 15B, 02-093 Warsaw, Poland

**Keywords:** Cancer, Computational biology and bioinformatics, Health care, Oncology

## Abstract

The purpose of this study was to estimate cancer survival in Poland between 2000 and 2019 for malignant neoplasms of female genital organs (FGO). We calculated survival in cancer of vulva, vagina, cervix uteri, corpus uteri, ovary, and other unspecified female genital organs. Data were obtained from the Polish National Cancer Registry. We estimated age-standardized 5- and 10-year net survival (NS) with the life table method and the Pohar-Perme estimator using the International Cancer Survival Standard weights. Overall, 231,925 FGO cancer cases were included in the study. The overall FGO age-standardized 5-year NS was 58.2% (95% confidence interval (CI) 57.9–58.5%) and the 10-year NS 51.5% (51.5–52.3%). Between 2000 and 2004 and 2015–2018, the highest statistically significant increase in age-standardized 5-year survival was noted for ovarian cancer at + 5.6% (P < 0.001). The FGO cancer median survival time was 8.8 years (8.6–8.9 years), with a standardized mortality rate of 6.1 (6.0–6.1), and with cause-specific years of life lost at 7.8 years (7.7–7.8 years). Hazard ratios (HR) increased with age at diagnosis (HR = 1.02, 95% CI 1.01–1.03, P = 0.001). Although FGO cancer survivorship has been consistently improving during the last twenty years, additional efforts need to be undertaken to improve survivorship in several FGO cancers.

## Introduction

Female genital organs (FGO) cancers affect millions of women of all ages globally. In 2020 alone, FGO cancers accounted for 16.0% of all cancer diagnoses and 15.3% of cancer-related deaths in women worldwide. Specifically, cervix uteri accounted for 7.8% and ovary 4.7%^1,2^ of all deaths. Cancer has an emotional, physical, material, and social impact on survivors and their families, in addition to having a significant economic cost^3–6^. The latest results from the Global Burden of Diseases, Injuries, and Risk Factors Study, covering the period 2000–2019, report FGO cancers as part of the leading cancers contributing to disability-adjusted life years, with cervical cancer occupying the fourth place, ovarian cancer the sixth, and corpus uteri the thirteenth place^7^.

Effective national cancer control strategies are required to address the rising cancer burden and accomplish the 2030 United Nations Sustainable Development Goals of reducing early mortality due to non-communicable diseases. In 2020, the Polish parliament adopted a new National Strategy for Oncology 2020–2030 to improve evidence-based prevention, population screening programs, effective treatment, and palliative care. The program strives to increase cancer survivorship and promote cancer monitoring through cancer registries.

Organized, comprehensive, and equitable implementation, backed by adequate funding, is the only way to turn national cancer control programs into measurable observations. Population-based cancer registries have grown in importance over the last half-century as a means of storing, presenting, and analyzing data for the prioritizing and tracking of cancer control efforts.

This study evaluated the current health situation of Polish female genital organ cancer patients and the time trends in their survival rates during the last two decades. We estimated cancer age-standardized relative survivals and other health metrics for FGO neoplasms, namely for vulva, vagina, cervix uteri, corpus uteri, ovary, and other unspecified FGOs. Together, these neoplasms accounted for more than 15% of all female malignant tumors in Poland in 2018. The two major FGO cancers contributing to this number were ovarian and corpus uteri cancers, which have, in the past twenty years, represented stable (corpus uteri) and decreasing (ovary) incidence and mortality rates.

## Materials and methods

### Source of data

Data were obtained from the Polish National Cancer Registry (PLCR), a non-profit national institution responsible for statistical and epidemiological cancer research in Poland (population 38.4 million in June 2019^8^). The cancer registry covers practically all incident cancer cases diagnosed in the Polish population. Data are actively collected from hospitals, healthcare practitioners, and palliative care centers. Reporting cancer to the PLCR is mandatory. All notifications are submitted by physicians and coded according to the International Statistical Classification of Diseases and Related Health Problems, Tenth Edition (ICD-10). At the PLCR, all cases are afterward verified by qualified coders based on histopathological/ cytological/ cytometry exam results and additionally coded applying the International Classification of Diseases for Oncology, Third Edition (ICD-O-3). In 2018, around 92% of registered cases were morphologically verified. Finally, the records are passed through specific tools to validate entries based on the valid causes of death in relation to the sex and age of the deceased. The PLCR system collection system is based on a unique Polish personal identification number (PESEL), which avoids double coding for the same patient.

### Identification of cancer cases

We conducted a cohort study including all Polish women diagnosed with malignant neoplasms of female genital organs (FGO), namely vulva (ICD-10 code C51); vagina (code C52); cervix uteri (code C53); corpus uteri (code C54); uterus, part unspecified (code C55), ovary (code C56), other and unspecified female genital organs—OUFGO (code C57), and placenta cancer (code C58), registered in the PLCR database between the 1st of January 2000 and the 31st of December 2018. Due to ICD-10 cause-of-death coding practices by physicians, we aggregated C55 into C54, henceforth retaining the name corpus uteri.

Vital status was verified until the 31st of December 2019, based on information obtained during the yearly controls of registered patients' vital status in the PESEL database. Exclusion criteria were as follows: (i) cases identified solely by death certificate, (ii) age at diagnosis below 15 years and over 99 years of age, (iii) individuals without personal identity number, or (iv) follow-up time below 30 days since diagnosis. Around 9 thousand cases were excluded from the analysis since they fulfilled at least one exclusion criterion (about 70%—criterion IV).

Since PLCR is a population-based registry that uses PESEL for registration and employs the PESEL database for yearly follow-up, there is no risk of loss to follow-up in the strict sense. Individuals who emigrated permanently from Poland and had no legal tie with the country may account for a meager portion of under-reported deaths. However, there is no reason to believe that these patients' cancer survival rates differed significantly and would introduce emigrant bias.

### Statistical analysis

Descriptive statistics are presented as mean and standard deviation (SD) or median and interquartile range (IQR) for numeric variables and numbers and percentages for categorical variables. Normality of continuous variables was assessed with the Jarque–Bera test or Shapiro–Wilk test, depending on the number of samples (below or above 5000 samples, respectively). Colormaps used Jenks natural breaks classification method (maximization of variance between classes and minimization within classes). Standardized mortality (SMR) ratios were standardized by age and year of diagnosis by indirect standardization. Patients were followed up from cancer diagnosis until death, the 31st of December 2019, or 10 years of observation, whichever occurred first.

We estimated age-standardized five and 10-year net survival (NS) deploying the life table method^9^ and the Pohar-Perme estimator^10^ for four periods of diagnosis, namely 2000–2004, 2005–2009, 2010–2014, and 2015–2018. The cohort approach was deployed, except for the periods where complete follow-up data were unavailable for all individuals, where the period analysis method^11^ was used. Age-standardized NS estimates were calculated using the International Cancer Survival Standard (ICSS) weights. Age at diagnosis was categorized into five age groups, namely 15–44, 45–54, 55–64, 65–74, and 75 + , following ICSS guidelines. The corresponding 95% confidence intervals (95% CIs) were estimated with log transformation. The between-group difference in time interval survivals was assessed with a two-tailed test. The associations were considered significant at the overall alpha level set at < 0.05.

Kaplan–Meier survival curves were produced to estimate survival rates at a specific time after diagnosis, and the corresponding 95% CIs were estimated with log transformation. Univariate and multivariate Cox proportional hazards regression models were fit to generate mortality hazard ratios (HR) and the corresponding 95% CIs, describing the association between exposures (age at the diagnosis and year of diagnosis) and time-to-event (death). Two-sided log-rank test was applied to assess the significance of exposures. HRs that include 1 in their 95% CI range were deemed not meaningful.

Cause-specific years of life lost (YLLs) were calculated using the age-specific life expectancy tables for the Polish population. The reference age was the average life expectancy for Polish women (between 1950 and 2019) at birth, namely 74.9 years, according to Statistics Poland^12^. Women diagnosed after this age were excluded from the YLLs analysis. YLLs' 95% CIs were produced using bootstrapping with 500 resamples from the cohort population. YLLs that include 0 in their 95% CI range were deemed not meaningful.

All statistical analyses were performed using RStudio Version 1.4.1103 (R Foundation for Statistical Computing, Vienna, Austria). Lifetables specific for the Polish population were obtained from Statistics Poland^12^.

### Compliance with ethical standards

According to Polish legislation, individual-level data from the PLCR can be used for statistics in aggregate form and scientific purposes. The need for ethics approval, written informed consent to participate in the study, is deemed unnecessary according to national regulations. Detailed legislative aspects of the National Polish Registry are regulated by Polish Law (Dz.U. 2018 poz. 1197). All analyses were carried out by relevant guidelines and regulations and are not classified as medical research involving identifiable human material or identifiable data. The PLCR obeys strict regulations to secure the complete confidentiality and protection of individuals. This study was conducted according to the Strengthening the Reporting of Observational studies in Epidemiology (STROBE) guidelines^13^.

## Results

### Patients’ characteristics

A total of 231,925 FGO neoplasm cases were included in the study. For the period 2000–2018 we included 8,732 cases of vulva cancer (ICD-10 C51); 1,819 of vagina cancer (ICD-10 C52); 58,099 of cervix uteri cancer (ICD-10 C53); 96,866 of corpus uteri cancer (ICD-10 C54 – C55), 64,461 of ovary cancer (ICD-10 C56), 1,948 of OUFGO (ICD-10 C57), and 175 of placenta cancer (ICD-10 C58). Due to the low number of placenta cancer cases, this cancer type was excluded from any statistical analysis. The detailed distribution of the number of cases by the period of diagnosis for each analyzed entity is presented in Table 1.

**Table 1:** Cases of female genital organs cancers by year of diagnosis, five and ten-year age-standardized net survival (cohort approach), age- and year-standardized mortality (SMR) ratios, and age-standardized cause-specific years of life lost (YLL) with respective 95% confidence intervals (95% CI)—Poland, 2000–2019.

	ICD-10 code	Number of cases					SMR (95% CI)	Median survival (95% CI) [years]	YLLs (95% CI) [years] τ = 74.9 years	Age-standardized net survival (95% CI)	
		Total	2000–2004	2005–2009	2010–2014	2015–2018				5-year	10-year
Vulva	C51	8732	1973	2151	2430	2178	5.0 (4.8–5.1)	3.4 (3.1–3.7)	6.1 (5.9–6.3)	54.5% (53.2–55.7%)	46.8% (45.1–48.4%)
Vagina	C52	1819	469	428	511	411	8.8 (8.3–9.3)	2.0 (1.8–2.2)	8.5 (8.2–8.9)	37.3% (34.8–39.8%)	29.9% (27.0–32.9%)
Cervix uteri	C53	58,099	17,209	16,373	14,381	10,136	9.7 (9.6–9.8)	6.5 (6.3–6.8)	11.6 (11.6–11.7)	55.4% (54.9–55.8%)	49.4% (48.8–50.0%)
Corpus uteri	C54–C55	96,866	20,053	24,204	27,887	24,722	3.1 (3.0–3.1)	*	3.0 (2.9–3.0)	75.4% (75.0–75.9%)	70.4% (69.7–71.1%)
Ovary	C56	64,461	15,383	16,746	17,706	14,626	12.1 (11.9–12.2)	3.7 (3.7–3.8)	11.2 (11.0–11.1)	39.8% (39.3–40.3%)	32.2% (31.5–32.9%)
OUFGO	C57	1948	455	503	542	448	11.5 (10.9–12.1)	1.7 (1.5–2.0)	9.0 (8.6–9.5)	38.5% (36.2–40.8%)	33.1% (30.3–36.0%)
Overall	C51–C57	231,925	55,542	60,405	63,457	52,521	6.1 (6.0–6.1)	8.8 (8.6–8.9)	7.8 (7.7–7.8)	58.2% (57.9–58.5%)	51.5% (51.5–52.3%)

*OUFGO* other and unspecified female genital organs.

*50% of the initial cohort population never achieved during the study period.

FGO cancers had a higher population direct-standardized incidence rate in central and southwestern Poland (74.0–77.9 cases per 100,000 women person-years) (Supplementary Fig. S1). For vulvar cancer, a higher incidence belt was observed in central Poland (2.9–3.2) while for cervix uteri in the northern and northwest regions (18.9–22.2). For corpus uteri and ovarian cancer, a lower incidence rate was observed in the north and northwest areas (0.0–29.1 and 0.0–19.2, respectively) (Supplementary Fig. S2).

**Figure 1:**
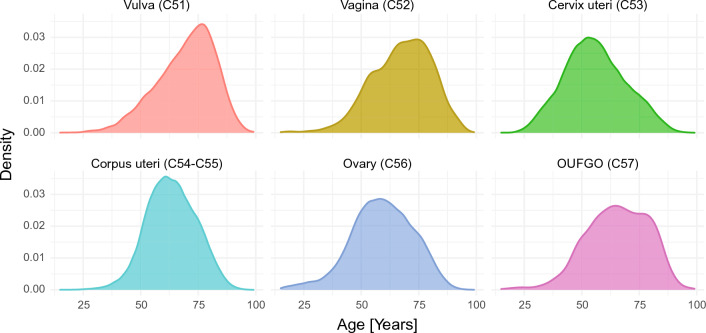
Kernel density estimates of age at diagnosis for malignant neoplasms of female genital organs, by cancer site (ICD-10 code)—Poland, 2000–2019. OUFGO – other and unspecified female genital organs.

The overall median age at diagnosis was 61 years (IQR = 18 years). The median age at diagnosis of vulva cancer was 71 years (17 years), vagina 69 years (18 years), cervix uteri 55 years (19 years), corpus uteri 63 years (15 years), ovary 59 years (19 years), and 66 years for OUFGO cancer (20 years) (Fig. 1).

The overall SMR for FGO was 6.1 (95% CI 6.0–6.1). The SMR for cancer of vulva was 5.0 (95% CI 4.8–5.1), vagina 8.8 (8.3–9.3), cervix uteri 9.7 (9.6–9.8), corpus uteri 3.1 (3.0–3.1), ovary 12.1 (11.9–12.2), and 11.5 (10.9–12.1) for OUFGO cancers (Table 1).

### Survival analysis for the time of the study

In the last period of analysis, i.e. 2015–2019, the overall age-standardized 5-year NS was 59.5% (95% CI 59.1–60.0%). For cancer of vulva was 53.8% (95% CI 51.6–55.9%), vagina 38.3% (34.0–42.6%), cervix uteri 56.9% (56.1–57.7%), corpus uteri 76.0% (75.4–76.7%), ovary 41.3% (40.4–42.1%), and for OUFGO cancer 39.0% (35.0–43.0%) (Table 1).

For the whole period of analysis, i.e. 2000–2019 the overall age-standardized 5-year NS was 58.2% (95% CI 57.9–58.5%). For cancer of vulva was 54.5% (95% CI 53.2–55.7%), vagina 37.7% (34.8–39.8%), cervix uteri 55.4% (54.9–55.8%), corpus uteri 75.4% (75.0–75.9%), ovary 39.8% (39.3–40.3%), and for OUFGO cancer 38.5% (36.2–40.8%) (Table 1). The overall age-standardized 10-year NS was 51.5% (95% CI 51.5–52.3%). For cancer of vulva was 46.8% (95% CI 45.1–48.4%), vagina 29.9% (27.0–32.9%), cervix uteri 49.4% (48.8–50.0%), corpus uteri 70.4% (69.7–71.1%), ovary 32.2% (31.5–32.9%), and for OUFGO cancer 33.1% (30.3–36.0%) (Table 1). Supplementary Fig. S3 represents the geographical (voivodship-level) distribution of the age-standardized 5- and 10-year NS for each cancer site.

The overall median survival time for FGO was 8.8 years (95% CI 8.6–8.9 years). For vulvar cancer it was 3.4 years (95% CI 3.1–3.7 years), vagina 2.0 years (1.8–2.2 years), cervix uteri 6.5 years (6.3–6.8 years), ovary 3.7 years (3.7–3.8 years), and 1.7 years (1.5–2.0 years) for OUFGO cancer. Corpus uteri cohort never achieved 50% of the initial cohort size during the observation period (Table 1).

The overall YLL for FGO for the whole analysis period was 7.8 years (95% CI 7.7–7.8 years). Specifically, for cancer of vulva 6.1 years (95% CI 5.9–6.3 years), vagina 8.5 years (8.2–8.9 years), cervix uteri 11.6 years (11.6–11.7 years), corpus uteri 3.0 years (2.9–3.0 years), ovary 11.2 years (11.0–11.1 years), and 9.0 years (8.6–9.5 years) for OUFGO cancer (Table 1).

### Survival analysis by year of diagnosis

**Figure 2:**
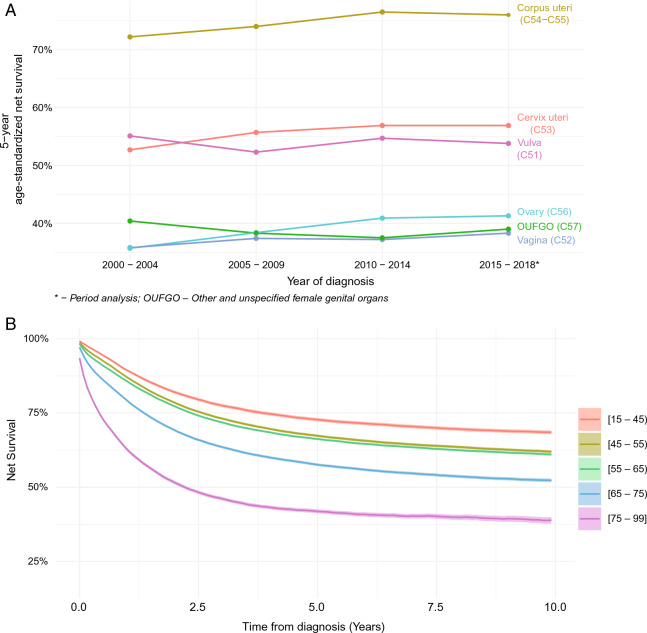
(**A**) Age-standardized five-year net survival by period of diagnosis and cancer site (ICD-10 code), and (**B**) Net survival by age-group for malignant neoplasms of female genital organs—Poland, 2000–2019.

The age-standardized NS has increased for almost all studied cancers between the first and last analysis period. The overall age-standardized 5-year NS has increased by 5.4 percentual points (pp) from 54.1 to 59.5% (P < 0.001). The highest statistically significant increase was noted for ovarian cancer (5.6 pp [from 35.7 to 41.3%, P < 0.001]) followed by cervix uteri cancer, with an increase of 4.2 pp (from 52.7 to 56.9%, P < 0.001) (Fig. 2A). No statistically significant decrease was observed during the study period. Detailed information on period-specific 5-year age-standardized NS and 95% CIs are presented in Table 2.

**Table 2:** Age-standardized five-year net survival and 95% confidence intervals (95% CI) for malignant neoplasms of female genital organs, by period of diagnosis—Poland, 2000–2019.

	ICD-10 code	Age-standardized 5–year net survival (95% CI)					
		2000–2004	2005–2009	2010–2014	2015–2018^†^	Difference 2000–2004 vs. 2015–2018^†^	
Vulva	C51	55.1% (52.5–57.7%)	52.3% (49.9–54.7%)	54.7% (52.4–56.9%)	53.8% (51.6–55.9%)	− 1.3% (− 1.6% to − 1.0%)	P = 0.280
Vagina	C52	35.8% (31.1–40.6%)	37.4% (32.3–42.5%)	37.2% (32.7–41.7%)	38.3% (34.0–42.6%)	+ 2.5% (+ 1.3% to + 3.7%)	P = 0.297
Cervix uteri	C53	52.7% (51.8–53.5%)	55.7% (54.8–56.5%)	56.9% (56.0–57.8%)	56.9% (56.1–57.7%)	+ 4.2% (+ 4.2 to + 4.2)	P < 0.001
Corpus uteri	C54–C55	72.2% (71.2–73.1%)	74.0% (73.2–74.8%)	76.5% (75.8–77.2%)	76.0% (75.4–76.7%)	+ 3.8% (+ 3.8% to + 3.8%)	P < 0.001
Ovary	C56	35.7% (34.7–36.7%)	38.4% (37.5 to 39.3%)	40.9% (40.0–41.7%)	41.3% (40.4–42.1%)	+ 5.6% (+ 5.6% to + 5.6%)	P < 0.001
OUFGO	C57	40.4% (35.6–45.1%)	38.3% (33.9–42.7%)	37.5% (33.4–41.6%)	39.0% (35.0–43.0%)	− 1.4% (− 2.5% to − 0.3%)	P = 0.364
Overall	C51–C57	54.1% (53.5–54.7%)	56.8% (56.3–57.4%)	59.7% (59.2–60.2%)	59.5% (59.1–60.0%)	+ 5.4% (+ 5.4 to + 5.4%)	P < 0.001

*OUFGO* other and unspecified female genital organs.

^†^period analysis.

While for all FGO cancers aggregated there was no statistically significant HR associated with the year of diagnosis (HR 1.00, 95% CI 0.98–1.01, P = 0.440), for ovarian cancer, the HR was statistically significant with a reduction of the risk with a later year of diagnosis (HR 0.98, 0.96–1.00, P = 0.018) (Supplementary Table S1).

### Survival analysis by age at diagnosis

Both 5- and 10-year NS decreased with age during the study period. In both intervals, 5- and 10-year NS, there was a survival plateau between the age groups of [15 – 45), [45 – 55), and [55 – 65), where a small decline in NS was observed (− 5.0 pp and − 1.1 pp for 5-year NS and − 6.4 pp and − 1.4 pp for 10-year NS, respectively), followed by a quick decrease in survival in the following age groups (− 8.3 pp and − 14.9 pp for 5-year NS and − 9.1 and − 13.8 pp for 10-year NS, for the age groups [65–75) and [75–99] respectively) (Table 2 and Fig. 2B). There was a statistically significant increased HR associated with age at diagnosis for the whole cohort (HR 1.02, 95% CI 1.01–1.03, P = 0.001) and specifically for vulva (HR 1.06, 95% CI 1.01–1.12, P < 0.024), corpus uteri (HR 1.04, 95% CI 1.02–1.06, P < 0.001), and ovary (HR 1.05, 95% CI 1.02–1.07, P < 0.001) cancers (Supplementary Table S1). Detailed information on age group and cancer type 5- and 10-year NS and 95% CIs is presented in Supplementary Table S2 and in Supplementary Fig. S4.

## Discussion

### Interpretation of the findings

Between 2000 and 2019, in the case of most FGO cancers, the survival of Polish patients has improved. The biggest statistically significant improvement was observed for ovarian cancer, followed by cancers of the cervix uteri and corpus uteri.

When comparing with the FGO survival leading countries in Europe, such as Norway and Finland, in the past decades, Poland has been approaching the survival values observed in these regions^14^. Comparing survival rates for the first and last time periods evaluated in this study (2000–2004 and 2015–2019) with the ones presented in the annual Nordic cancer registries’ reports, the gap between countries for FGO cancers (Finland only) has reduced from − 10.8 to − 6.0% points (pp) in less than twenty years of observations^15,16^. However, Poland has lagged behind Norway, with the most significant difference (3.9%) in ovarian and OUFGO cancers age-standardized NS^17^. When comparing age-standardized NS between Poland and Nordic countries, the most significant difference was observed for cervical cancer (− 16.2 pp compared with Finland and − 25.1 pp when compared with Norway) (Supplementary Table S3). The observed Polish survival rates are closer to those of neighboring and former communist bloc countries (from − 22.2% in NS for vulvar cancer in Germany and -10.1% for cervical cancer in Estonia to + 0.5% for ovarian cancer in Czechia)^18–20^. This also accords with our earlier observations^21^, where we argue that these differences can be attributed with disparities in cervical cancer screening attendance rates between countries, leading to impacts in the effectiveness of the national screening program, lack of access to HPV vaccines through the Polish Childhood Immunization Program, or related to the still unaligned healthcare gaps of the former communist bloc countries. Apart from Poland, vaccination against HPV is freely available as part of the girls’ national immunization strategies in all European Union countries, either fully or partly funded by the states^22^. Therefore, if Poland aims to fulfill the WHO Global strategy program to eliminate cervical cancer as a public health problem^23^, a key Polish policy priority should be the long-term improvement of the existing screening program with a high-performance test by 35 years of age and again by 45 years of age and incorporating HPV vaccination into the national vaccination program for girls up to 15 years of age.

While the factors mentioned above, namely differences in cancer screening attendance rates, HPV vaccination coverage, or healthcare services access and quality, can potentially explain the spatial distribution disparity of population-normalized incidence rates for cervical cancer at the voivodship level, they cannot explain the survival distribution of other high incidence FGO cancers, that is ovarian and corpus uteri cancer. Therefore, future studies should focus on finding the potential causes for the different spatial distributions of these cancers, including demographic, social, economic, habitual, and environmental factors.

Another explanatory variable that might clarify the differences in survival between countries is the health care expenditure (HCE), measured as the declared percentage of gross domestic product attributed to HCE (GDP-HCE). The GDP-HCE englobes not only cancer screening or treatment but it serves as a broader proxy of investments in oncological-related healthcare. According to the GDP-HCE data provided by the statistical office of the European Union, covering the 2013–2019 period, Poland has slightly increased its expenditure from 6.41 to 6.45% (+ 0.04 pp), while other countries, such as Norway, Germany, or Estonia, have increased it relatively more, that is from 8.93% to 10.54% (+ 1.61 pp), from 11.00% to 11.70% (+ 0.70 pp), and from 6.02% to 6.82% (+ 0.80 pp), respectively. Exceptions were Finland and Czechia, which, in the same period, reduced their GDP-HCE from 9.81% to 9.17% (− 0.64 pp) and from 7.72% to 7.60% (− 0.12 pp), respectively. One can notice at first glance that countries with a higher GDP-HCE tend to be characterized by higher survival rates in the case of FGO cancers. However, any potential hypothesis regarding the link between HCE and cancer survival must be taken lightly since the country's involvement in oncological-related services is not linearly associated with its HCE, and there are many other related factors^24^.

The highest SMR was observed for ovarian and OUFGO (12.1 and 11.5 respectively) cancers, two cancer sites that were often grouped in previous studies^25–29^, and the lowest for corpus uteri cancers (SMR = 3.1). These results match those observed in previous studies, where ovarian cancer is the most lethal gynecological tumor^3,30,31^. Compared with the latest international survey on the estimates of mortality for cervical cancer, our study presents a higher SMR (9.7 vs. 4.9)^32^. Even if the publication by Arbyn et al. is based on estimates and not real data, since it represents the most up-to-date international analysis, an assessment of the correspondence of our findings is worthwhile. To do so, we calculated the SMR for 2018 using PLCR data obtaining 7.57 (95% CI 7.19–7.97). Even if still higher than Arbyn et al. estimates, this value is closer to the SMR values presented by the other Eastern European countries (3.8 in Belarus to 8.9 in Romania). This also broadly aligns with previous works regarding other Eastern European countries. In Hungary, for the period 1979–2013, the reported SMR was 6.9 for cervical cancer (9.7 in Poland), 6.5 for uterus cancer (3.1 in Poland), and 7.4 for ovarian cancer (12.1 in Poland)^33^. Even if the higher SMRs for Poland depicts a lower survival due to several potential factors (i.a. screening coverage, vaccination), a note of caution is due when comparing SMRs from different studies due to different standardization methods and “standard” populations employed in the calculations^34–37^, periods under study, national coverage of cases, or comparing estimates with real-data from cancer registries, for example. Finally, our findings are derived from a specific nationwide cohort, and generalizations to other countries should be made with caution.

When analyzing specific cancers median survival times, Poland still presents lower values to the other European countries, with the most significant differences in OUFGO cancers (1.7 years in Poland [2000–2019], 4.3 for Belgium [2000–2008, and 5.0 years in Norway [2019])^17,38,39^, potentially due to coding differences between countries. The median survival time for all FGO-aggregated cancers for Poland was 8.8 years. Comparison to neighboring countries was not possible due to the lack of previously published reports that would allow for median survival analysis comparison, either by reported values or graphical inference from survival curves.

Between 2000 and 2019, there were 1,772,768 YLLs attributable to FGO cancers in Poland. Ovarian and cervix uteri cancers accounted together for 1,395,912 YLLs (79% of all FGO YLLs under analysis), followed by corpus uteri cancer with 290,598 YLLs (16%). Cervical cancer (673,948 YLLs, 38%), as the only FGO cancer that can be covered by primary and secondary prevention, should be a target for healthcare policymakers.

### Suggestions for the future research work

Further work is required to establish the origins of Poland's unsatisfactory FGO cancer survival rates. To develop a complete picture of FGO cancer survival, additional studies will be needed to assert the influence of demographical, social, economic, environmental variables, as also stratifying the analyses by cancer staging and morphology, all factors that could potentially affect cancer survival. Only such comprehensive analysis can help policymakers and stakeholders target the required reforms needed to curtail the existing health chasm between European countries. Furthermore, future research on YLLs due to FGO cancers could deploy different YLL metrics, such as the standard expected years of life lost, to allow for international comparisons and for studying entire cohorts without country-specific maximum age threshold (life expectancy). Additionally, there is the need to investigate the influence of the COVID-19 pandemics in cancer survival due to the temporary limitations in admittance to beforehand planned therapies, delayed diagnosis, and privately held cervical cancer screening routines. Prior studies have shown that, during pandemics, Polish population cancer screening programs are characterized by a lower participation rate than in the pre-pandemic period^40^. Furthermore, due to the hosting of over a million Ukrainian women in Poland (state for 20th of March 2022), it is expected that the incidence, prevalence, and survival of FGO cancers will shift, e.g. as a result of two-fold higher cervical cancer incidence in Ukrainian women^41^. Nonetheless, the effects of the pandemics and refugees’ influx will only be seen in decades.

### Limitations and strengths of the study

One limitation of our study was that, although our cohort was large, the number of patients in some subgroups was small, namely for placental cancer. This made it impossible to calculate survival analysis and other time-dependent measures, which is a usual limitation in analyzing rare cancers.

The main strengths of this study were the large study population and the complete national coverage of cancer cases, ensuring a high representativity and generalizability of the study results. Furthermore, since the Polish healthcare system is subsidized by governmental institutions, access to both diagnostic and treatment measures is equal regardless of socioeconomic status, excluding potential biases due to healthcare services’ unaffordability.

Within the study's duration, i.e. between 2000 and 2019, the percentage of morphologically verified cancer cases registered by PLCR increased from approximately 65–92%. The mortality to incidence ratio changed from around 0.7–0.5 in the same time frame. Both metrics depict an improvement in cancer registration and data quality. It is reasonable to suppose that the unreported cases from twenty years ago had a lower survival rate than those in our report. As a result, the observed improvements in cancer survival over the last two decades may be underestimated.

## Conclusions

In the last two decades, the survival of the Polish FGO cancer patients has been consistently improving. The highest increase in age-standardized 5-year NS was observed for ovarian and cervical cancer, while the lowest for vulvar and OUFGO cancers. Although FGO cancer survivorship has been consistently improving during the last twenty years, additional efforts need to be undertaken to improve survivorship in several FGO cancers.

## Supplementary Information

Supplementary Information.

## Data Availability

The data analyzed in this study was obtained from the Polish National Cancer Registry and is available upon reasonable request and subject to the ethical approvals in place and material transfer agreements.
