# Supplementary material for: Survival of patients with cancers of the female genital organs in Poland, 2000–2019
Source: Sci Rep. 2023 May 25;13:8473. doi: 10.1038/s41598-023-35749-6 (PMC10209933; doi:10.1038/s41598-023-35749-6)
Supplement: Supplementary file 1 — Supplementary Information. [file 41598_2023_35749_MOESM1_ESM.pdf]

Supplementary Figure S1. Population normalized incidence rate (per 100,000 person-years) for malignant neoplasms of female genital organs, by voivodship - Poland, 2000-2019

OUFGO – Other and unspecified female genital organs.

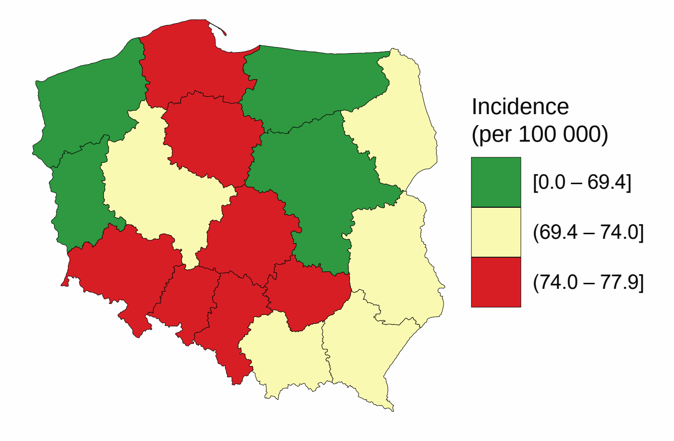

Supplementary Figure S2. Population-normalized incidence rate (per 100,000 person-years) for malignant neoplasms of female genital organs, by cancer site (ICD-10) and voivodship - Poland, 2000-2019. OUFGO – Other and unspecified female genital organs.

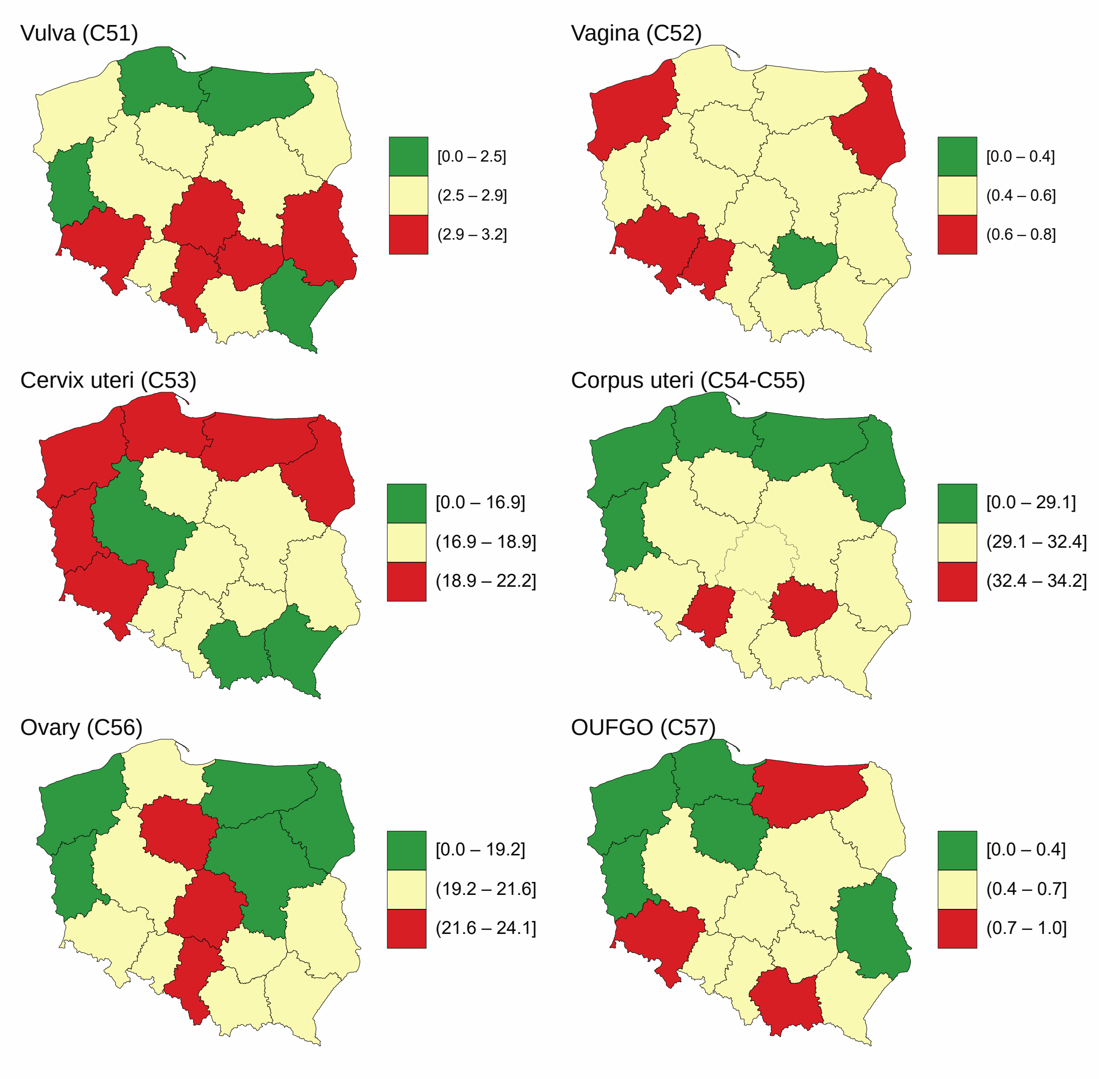

Supplementary Figure S3. Age-standardized 5-year and 10-year net survival rates, by cancer site (ICD-10 code) and voivodship - Poland, 2000 – 2019.

OUFGO – Other and unspecified female genital organs.

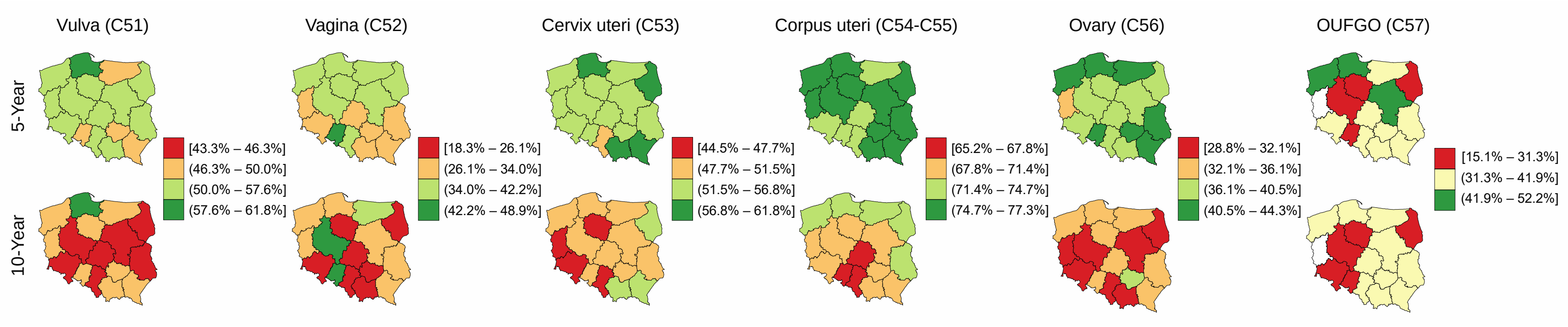

Supplementary Figure S4. Net survival for malignant neoplasms of female genital organs by cancer site (ICD-10 code) and by age-group - Poland, 2000-2019.

OUFGO – Other and unspecified female genital organs.

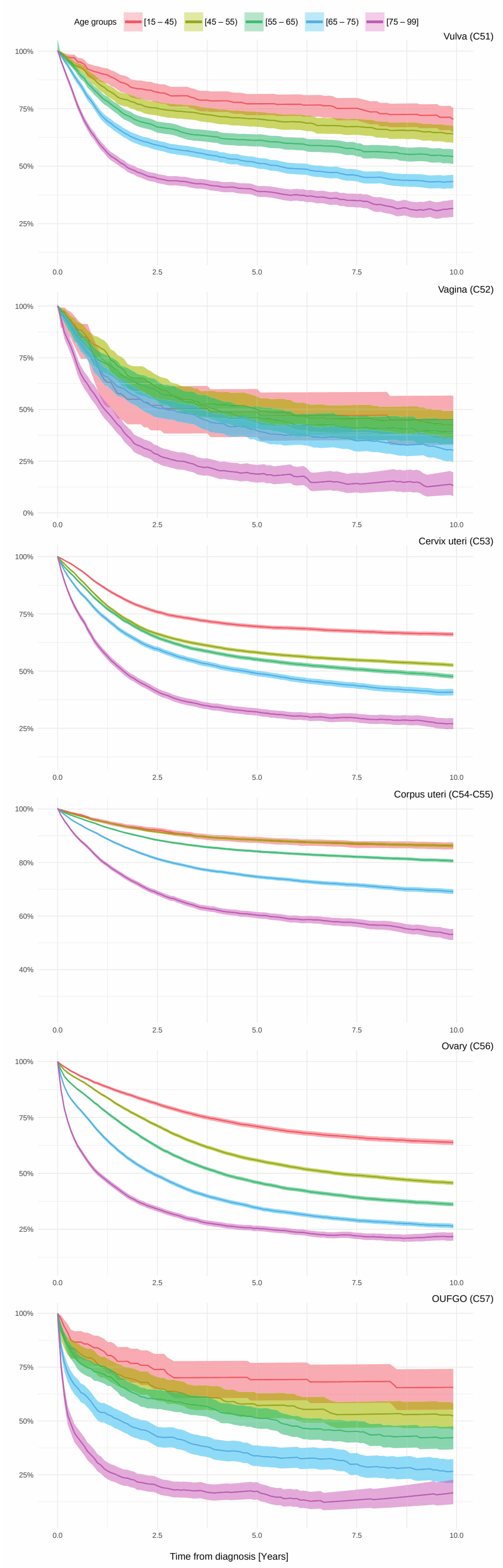

Supplementary Table S1. Mortality hazard ratios and 95% confidence interval (95% CI) by cancer site (ICD-10 code) - Poland, 2000 – 2019.

		Hazard ratio (95% CI)			
		Univariate		Multivariate	
	ICD-10 code	Year of diagnosis	Age at diagnosis	Year of diagnosis	Age at diagnosis
Vulva	C51	1.00 (1.00 to 1.01) P = 0.292	1.05 (1.04 to 1.05) P < 0.001	0.98 (0.93 to 1.03) P = 0.379	1.06 (1.01 to 1.12) P = 0.024
Vagina	C52	0.99 (0.98 to 1.00) P = 0.260	1.04 (1.03 to 1.04) P < 0.001	1.02 (0.92 to 1.14) P = 0.778	1.00 (0.90 to 1.12) P = 0.895
Cervix uteri	C53	1.00 (0.99 to 1.00) P = 0.006	1.03 (1.03 to 1.03) P < 0.001	1.02 (1.00 to 1.04) P = 0.103	1.00 (0.98 to 1.03) P = 0.797
Corpus uteri	C54 – C55	0.99 (0.99 to 0.99) P < 0.001	1.07 (1.07 to 1.07) P < 0.001	1.01 (0.98 to 1.03) P = 0.635	1.04 (1.02 to 1.06) P < 0.001
Ovary	C56	0.99 (0.99 to 0.99) P < 0.001	1.04 (1.04 to 1.04) P < 0.001	0.98 (0.96 to 1.00) P = 0.018	1.05 (1.02 to 1.07) P < 0.001
OUFGO	C57	1.01 (1.00 to 1.02) P = 0.064	1.05 (1.05 to 1.06) P < 0.001	1.00 (0.89 to 1.11) P = 0.946	1.05 (0.94 to 1.17) P = 0.352
**Overall**	**C51 – C57**	**0.99 (0.99 to 0.99) P < 0.001**	**1.04 (1.04 to 1.04) P < 0.001**	**1.00 (0.98 to 1.01) P = 0.440**	**1.02 (1.01 to 1.03) P = 0.001**

OUFGO – Other and unspecified female genital organs

Supplementary Table S2. 5-year and 10-year net survival rates with 95% confidence intervals (95% CI) by cancer site (ICD-10 code) and age-group - Poland, 2000 – 2019

		Net survival (95% CI)									
		5-Year					10-Year				
	ICD-10	[15 – 45)	[45 – 55)	[55 – 65)	[65 – 75)	[75 – 99]	[15 – 45)	[45 – 55)	[55 – 65)	[65 – 75)	[75 – 99]
Vulva	C51	77.1% (72.0% to 81.3%)	70.6% (67.2% to 73.7%)	61.2% (58.6% to 63.8%)	51.8% (49.5% to 54.1%)	39.7% (37.3% to 42.0%)	70.3% (64.6% to 75.4%)	64.0% (60.1% to 67.6%)	54.1% (50.9% to 57.2%)	43.3% (40.4% to 46.2%)	31.6% (27.9% to 35.4%)
Vagina	C52	48.8% (36.7% to 59.9%)	50.5% (43.7% to 56.8%)	46.1% (40.6% to 51.5%)	40.5% (35.6% to 45.3%)	18.9% (14.8% to 23.4%)	45.1% (32.8% to 56.7%)	42.6% (35.6% to 49.3%)	39.4% (33.3% to 45.5%)	30.2% (24.6% to 36.0%)	13.2% (8.1% to 19.6%)
Cervix uteri	C53	69.6% (68.7% to 70.5%)	58.3% (57.5% to 59.1%)	55.2% (54.4% to 56.1%)	49.2% (48.0% to 50.4%)	32.3% (30.7% to 33.9%)	66.1% (65.2% to 67.0%)	52.6% (51.7% to 53.4%)	47.7% (46.7% to 48.6%)	40.8% (39.3% to 42.2%)	27.0% (24.6% to 29.4%)
Corpus uteri	C54 – C55	88.5% (87.3% to 89.6%)	88.5% (88.0% to 89.1%)	84.2% (83.8% to 84.7%)	74.8% (74.2% to 75.5%)	60.5% (59.3% to 61.7%)	86.3% (84.9% to 87.6%)	86.3% (85.6% to 86.9%)	80.6% (80.0% to 81.2%)	69.1% (68.2% to 70.1%)	53.2% (51.1% to 55.2%)
Ovary	C56	71.2% (70.2% to 72.2%)	56.1% (55.3% to 57.0%)	46.2% (45.4% to 47.0%)	34.9% (34.0% to 35.8%)	25.4% (24.2% to 26.6%)	63.8% (62.7% to 65.0%)	45.7% (44.8% to 46.6%)	36.2% (35.3% to 37.1%)	26.4% (25.4% to 27.4%)	21.8% (19.9% to 23.7%)
OUFGO	C57	69.2% (59.4% to 77.0%)	58.0% (51.9% to 63.8%)	51.7% (46.8% to 56.3%)	33.8% (29.0% to 38.6%)	17.2% (13.4% to 21.4%)	65.6% (55.2% to 74.2%)	52.3% (45.9% to 58.4%)	42.3% (36.8% to 47.6%)	26.6% (21.2% to 32.3%)	16.6% (11.4% to 22.8%)
**Overall**	**C51 – C57**	**72.9% (72.3% to 73.5%)**	**67.9% (67.5% to 68.3%)**	**66.8% (66.4% to 67.2%)**	**58.5% (58.0% to 59.0%)**	**43.5% (42.8% to 44.3%)**	**68.3% (67.6% to 68.9%)**	**61.8% (61.4% to 62.3%)**	**60.5% (60.0% to 60.9%)**	**51.4% (50.7% to 52.0%)**	**37.5% (36.3% to 38.7%)**

OUFGO – Other and unspecified female genital organs

Supplementary Table S3. Comparison of 5-year net survival rates by cancer site (ICD-10 code) - Poland and other European countries, 2000 – 2019

		5-Year Net survival (95% CI)					
		FGO overall	Vulva C51	Cervix uteri C53	Corpus uteri C54	Ovary C56	Ovary, OUFGO C56 - C57
Finland[16]	2002 – 2004	64.9% (63.1% to 66.6%)	-	74.9% (71.1% to 78.9%)	81.0% (78.5% to 83.5%)	-	43.2% (40.5% to 46.0%) *
	2017 – 2019	65.5% (64.1% to 67.0%)	-	73.1% (69.3% to 77.1%)	81.5% (79.6% to 83.6%)	-	45.2% (42.8% to 47.8%) *
Norway[17]	2001 – 2005	-	-	77.6%	81.6%	-	41.9%*
	2016 – 2020	-	-	82.0%	85.6%	-	51.1%*
Germany[20]	2017 - 2018	-	76.0%	63.0%	78.0%	42%	-
Czechia[18]	2014 - 2018	-		63.1% (61.6% to 64.5%)	80.6% (79.5% to 81.6%)	40.8% (39.4% to 42.2%)	
Estonia[19]	2012 - 2016	-		67.0%	80.0%	47.0%	
Poland	2000 – 2004	54.1% (53.5% to 54.7%)	55.1% (52.5% to 57.7%)	52.7% (51.8% to 53.5%)	72.2% (71.2% to 73.1%)	35.7% (34.7% to 36.7%)	35.8% (34.8% to 44.4%)
	2015 – 2018	59.5% (59.1% to 60.0%)	53.8% (51.6% to 55.9%)	56.9% (56.1% to 57.7%)	76.0% (75.4% to 76.7%)	41.3% (40.4% to 42.1%)	41.1% (40.3% to 41.9%)
Difference	Finland	-10.8%	-	-22.2%	-8.8%	-	-7.4%
		-6.0%	-	-16.2%	-5.5%	-	-4.1%
	Norway	-	-	-24.9%	-9.4%	-	-6.1%
		-	-	-25.1%	-9.6%	-	-10.0%
	Germany	-	-22.2%	-6.1%	-2.0%	-0.7%	-
	Czechia	-	-	-6.2%	-4.6%	+0.5%	-
	Estonia	-	-	-10.1%	-4.0%	-5.7%	-

* – C56, C57.0-4, C48.1-2
OUFGO – Other and unspecified female genital organs
CI – confidence interval
